# The development and utility of a multicriteria patient decision aid for people contemplating treatment for osteoarthritis

**DOI:** 10.1111/hex.13505

**Published:** 2022-08-30

**Authors:** Sam G. Moreton, Glenn Salkeld, Sally Wortley, Yun‐Hee Jeon, Hema Urban, David J. Hunter

**Affiliations:** ^1^ School of Psychology, Faculty of the Arts, Social Sciences and Humanities University of Wollongong Wollongong New South Wales Australia; ^2^ Faculty of the Arts, Social Sciences and Humanities University of Wollongong Wollongong New South Wales Australia; ^3^ Consumer Evidence and Engagement Unit Australian Department of Health Sydney New South Wales Australia; ^4^ Sydney Nursing School, Faculty of Medicine and Health The University of Sydney Sydney New South Wales Australia; ^5^ Rheumatology Department, Institute of Bone and Joint Research, The Kolling Institute, Royal North Shore Hospital The University of Sydney Sydney New South Wales Australia; ^6^ The Lancet Regional Health Elsevier Sydney Australia

**Keywords:** decision aid, decision support tool, multicriteria decision analysis, osteoarthritis, osteoarthritis treatment, treatment decision‐making

## Abstract

**Background:**

There are a range of treatment options for osteoarthritis (OA) of the knee and hip, each with a unique profile of risks and benefits. Patient decision aids can help incorporate patient preferences in treatment decision‐making. The aim of this study was to develop and test the utility of a patient decision aid for OA that was developed using a multicriteria decision analytic framework.

**Methods:**

People contemplating treatment for OA who had accessed the website myjointpain.org.au were invited to participate in the study by using the online patient decision aid. Two forms of the patient decision aid were created: A shorter form and a longer form, which allowed greater customization that was offered to respondents after they had completed the shorter form. Respondents also completed questions asking about their experience using the patient decision aid.

**Results:**

A total of 625 self‐selected respondents completed the short‐form and 180 completed the long‐form. Across both forms, serious side effects, pain and function were rated as the most important treatment outcomes. Most respondents (64%) who completed the longer form reported that using the tool was a positive experience, 38% reported that using the tool had changed their mind and 48% said that using the tool would improve the quality of their decision‐making.

**Conclusions:**

Overall, the findings suggest that this patient decision aid may be of use to a substantial number of people in facilitating appropriate treatment decision‐making.

**Patient or Public Contribution:**

Service users of myjointpain.org.au were involved through their participation in the study, and their feedback will guide the development of future iterations of the tool.

## 1 INTRODUCTION

Amongst the different medical, pharmacological, surgical and lifestyle options for the treatment of osteoarthritis (OA), the choice of ‘what's best’ can be difficult to navigate as a patient. Each treatment option has a different profile of potential benefits, harms, risks and costs. What is best for an individual patient is a matter of personal preference. This is where patient decision aids (PDAs) can help. PDAs are tools used to facilitate a patient's understanding of the pragmatic tradeoffs inherent to each treatment, and are aimed at helping facilitate patient deliberation in consultation with their treatment team. Substantial research supports PDAs as effective in improving patient knowledge, reducing decision conflict and strengthening treatment choice.^1^ Patients tend to be happy to use PDAs, and a high proportion follow through with their decision.^2^ Clinicians also appear to be open to the use of PDAs, with Adams et al.^3^ reporting that 79% of orthopaedic consultants consider the use of PDAs to be a ‘good’ or ‘excellent’ idea. PDAs have also been shown to improve orthopaedic surgeons' satisfaction and consultation efficiency.^4^ Implementing shared decision‐making practices has many benefits such as improved decision quality, increased patient satisfaction and economic benefits through the reduction of unnecessary procedures.^5^ Although patient agency is integral to shared decision‐making, the overwhelming majority of patients prefer decisions made *in conjunction* with their physician.^6^

To this end, numerous PDAs have been developed for the treatment of OA.^4^, ^7^, ^8^, ^9^, ^10^, ^11^, ^12^, ^13^, ^14^, ^15^, ^16^, ^17^, ^18^, ^19^, ^20^, ^21^, ^22^, ^23^, ^24^, ^25^ However, the existing PDAs for OA tend to have the following limitations. First, many existing PDAs cannot be personalized to the patients' clinical characteristics or capture their preferences for the attributes of treatment and consequent benefits, risks and potential harms. This impersonalization leads to the inclusion of general, possibly redundant information that may not address what is important to the patient as the decision‐maker. Few PDAs will guide the patient through the process of deciding what is best for them by integrating personal preferences with probabilistic evidence of benefits, risk and harm. Instead, many PDAs^12^ require patients to complete the cognitively demanding task of weighing up the risks and benefits of each treatment and intuit the optimal treatment. These PDAs stop short of combining any quantitative estimate of preferences gleaned from the values clarification exercise with the quantitative estimate of effect. As a consequence, the patient is not given any information on the rank order of treatment options based on the expected value of the outcome (the summed total of the probability of effect multiplied by the importance weight derived for each attribute of treatment). It is unrealistic to expect patients to perform the mathematical operation needed to generate the expected value for each treatment option. Yet arguably, both intuition and analysis are required for good decision‐making.

## 2 OBJECTIVE OF THIS PAPER

This study was part of a larger study into the effects of the Osteoarthritis Awareness Hub.^26^ Incorporating feedback from users is integral to developing decision support systems that best suit the needs of these populations. The main objective of this study was to test the following questions: First, is an online multicriteria PDA useful for people with OA who are considering different treatments? Second, how might the usefulness of the PDA differ across age groups? Third, which treatment attributes were considered most important to respondents?

## 3 METHODS

### 3.1 Developing the tool

A personalized OA PDA was developed that combined patient preferences and current clinical evidence to provide a numerical ‘score’ for treatment options relevant to the patient. The Annalisa (AL) decision support tool utilizes a simple weighted‐sum approach, whereby user‐determined preference weights for treatment outcomes are multiplied by the performance of treatments on these outcomes to provide an estimate of the value of each potential treatment. This approach was adopted for the purpose of an online decision aid as it is quick to compute the expected value (as opposed to more time‐consuming methods for determining preference weights such as the analytical hierarchy process), and the AL system allows transparency, whereby users can see how the changing of preference weights changes the treatment recommendations.

### 3.2 Attribute identification (patient‐important outcomes)

To determine the attributes to be used in the OA decision tool, a systematic review of the qualitative and preference‐based literature was undertaken. The focus of the literature review was to identify studies that included individuals with OA (or suspected OA) of the hip and knee, and included information on values, preferences or factors (‘attributes’) that are important to these individuals when making treatment decisions. A set of 12 patient important outcomes was identified following the literature review: reduction in pain, improvement in function and stiffness, avoidance of side effects (mild and serious), invasiveness, time burden, duration of action, out‐of‐pocket expenses, recovery time, emotional well‐being and duration of pain relief. Following discussions with the research team, this was reduced to nine outcomes. This was based on the opinion that the estimates underpinning the three outcomes (recovery time, emotional well‐being, duration of pain relief) were not as relevant to all OA patients and/or estimates would be difficult to identify. Further details of the systematic review protocol can be found in Appendix SA.

### 3.3 Treatment options

Based on the options described on the myjointpain.org.au website, 20 treatment options were included in the OA decision tool. A second review of the literature was undertaken to identify the best available evidence for each of these 20 treatment options. Emphasis was placed on identifying evidence from clinical guidelines, systematic reviews, meta‐analyses or health technology assessment reports. The Osteoarthritis Research Society American Academy of Surgeons Clinical Practice Guidelines on Treatment of Osteoarthritis of the Knee were used as the main sources of evidence. Additional search identified a further 51 studies relevant to the OA AL (see Appendix SA). These additional studies covered areas not reported on in the above guidelines. Relevant data points were extracted and presented to the research team for discussion. A final spreadsheet was created with all the evidence points.

### 3.4 Personalizing the DA

Within the Annalisa DA, there is a capacity to personalize the options and preferences for that individual user. This includes the choice to select which attributes to include in the DA and utilize the probability ratings for each of the attributes (benefits and potential harms) that are stratified by the respondent's own personal and clinical characteristics. The greater the level of personalization, all things being equal, the greater the time and effort required by the patient to complete the DA and get the most out of the results. Not all users will want or even need that level of personalized decision support. For this reason, we created two versions of the DA: a ‘fast and frugal’ (short form) version with a minimal level of personalization, along with a longer and customizable version that provided higher levels of personalized decision support.

### 3.5 Short‐form DA

**Figure 1:**
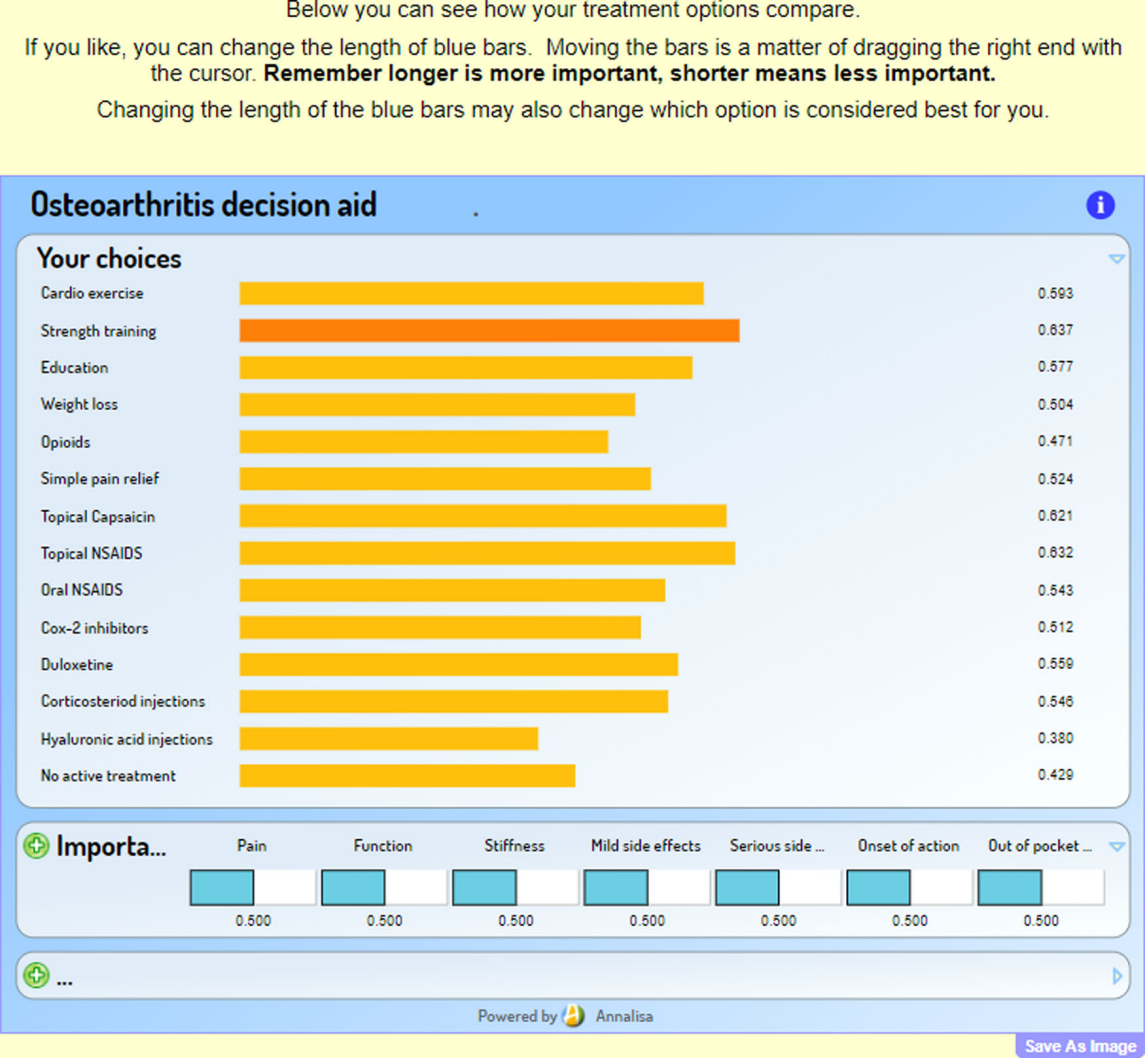
Screenshot of the short‐form Annalisa decision aid

Respondents first landed on a page that contained a link to the Participant Information Sheet and provided an explicit description of the decision and the health problem in question (treatment decisions for knee and/or hip OA). Respondents then selected what sorts of treatments they were interested in: (1) lifestyle changes and medicines; (2) medicines and surgery; (3) lifestyle changes and surgery; or (4) medicines only. These combinations were chosen to limit the cognitive load on the respondents, and these choices limited the options in the PDA to focus on those treatments that interested the respondents. Respondents then weighted on a 10‐point scale the importance of seven fixed attributes: pain, function, stiffness, mild side effects, serious side effects, the onset of action and out‐of‐pocket costs (values clarification). These scores were inputted into the short‐form Annalisa as the attribute weights. Respondents then moved onto the short‐form decision aid itself. Here, they could see the scores that they received for each treatment option (see Figure 1). They could further modify the weight of the attributes by sliding the blue bar for each attribute, thus changing the score.

After moving on from the short‐form decision aid, respondents then completed three outcome questions: (1) they rated how surprised they were with the option that emerged with the highest score on a five‐point scale from ‘not at all’ to ‘extremely’; (2) they selected ‘yes’ or ‘no’ as to whether the decision aid changed their views about treatment; and (3) they rated how likely (‘very likely’, ‘likely’, ‘not likely’) it would be that they would visit their general practitioner (primary care physician) or specialist in the next 6 months to talk about their treatment options.

### 3.6 Long‐form

After completing the short form, respondents had the option to continue to a longer version of the PDA. Respondents first watched a short video featuring one of the authors explaining the research. Respondents then provided basic demographics such as age, which joints were most affected and comorbidities. Similar to the short form, respondents then selected the treatments that they would be interested in: (1) lifestyle options and devices; (2) medicines; (3) alternative medicines or complementary therapies; and (4) surgical interventions. Unlike in the short form, respondents were not forced to select particular combinations of therapies (e.g., lifestyle options and medicines) but could select any number of the four treatment categories. This was then further customized: respondents could then choose specific treatments in each category that they were interested in. For instance, if they chose lifestyle options, respondents could select ‘yes’ or ‘no’ to a range of treatment options (e.g., cardiovascular training, strength training, walking canes). For each treatment, patients had the option of clicking a link to a website (myjointpain.org.au) that explained the treatment in more depth. Following this, patients selected (yes/no) which treatment attributes (e.g., relieving pain, improving function) were essential to them in comparing their treatment options. Attributes that were not selected were not included in the DA. Respondents next rated how time‐consuming and invasive they thought certain types of treatments would be. The reasoning behind this is that there are individual differences in how patients perceive treatments to vary on these outcomes. For instance, one person who is very busy during the day may find treatments where they need to apply a cream regularly to be very intrusive, whereas a person who is retired might not consider it to be so intrusive. Respondents then landed on a page that had instructions for how to use the tool, including a short instructional video.

There were then three instances of the personalized DA. A first Annalisa DA required the respondents to set the weights of the attributes by sliding the blue bars from a default of 0.5 (see Figure 1). The second instance then showed the respondents their treatment scores and gave them the option to modify their weights further by again sliding the blue bars. The third instance was optional for patients who wanted to see the underlying evidence behind each treatment for each attribute. The ability for respondents to view the underlying numerical ratings estimate of the performance of each treatment on each outcome is a way in which the decision aid fulfills the criteria of providing information on options and their benefits and harms. Respondents could again modify the weights if they wished.

Respondents then answered four questions about how easy the tool was to use; these were scored from ‘very hard’ to ‘very easy’. Respondents then answered how surprised they were by the result, whether it had changed their views on their treatment options, the likelihood they would visit their GP (‘very likely’, ‘likely’, ‘not likely’) and whether they thought the experience of using the decision aid would help them improve decision quality (*yes*, *no*, *unsure*).

### 3.7 Distribution of the online survey

The decision aid was placed on the myjointpain.org.au website in September 2015 and was advertised through the Arthritis Australia newsletter and Facebook pages. The study received approval from the University of Sydney Human Research Ethics Committee. The only requirement for participation was that respondents must have knee and/or hip OA and be considering their treatment options.

### 3.8 Analysis plan

**Figure 2:**
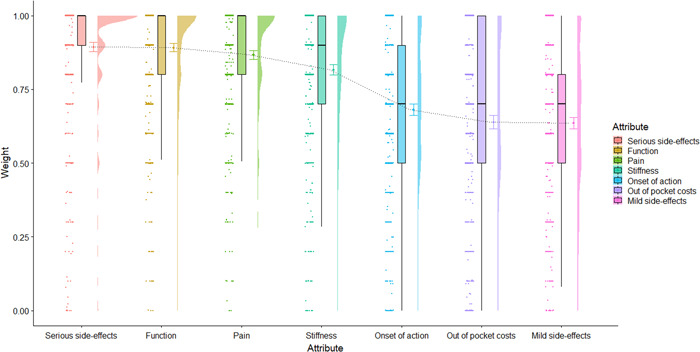
Raincloud plots of short‐form respondents determined importance weights for treatment attributes (*N* = 700). Higher weights indicate higher importance given to the respective attribute

**Figure 3:**
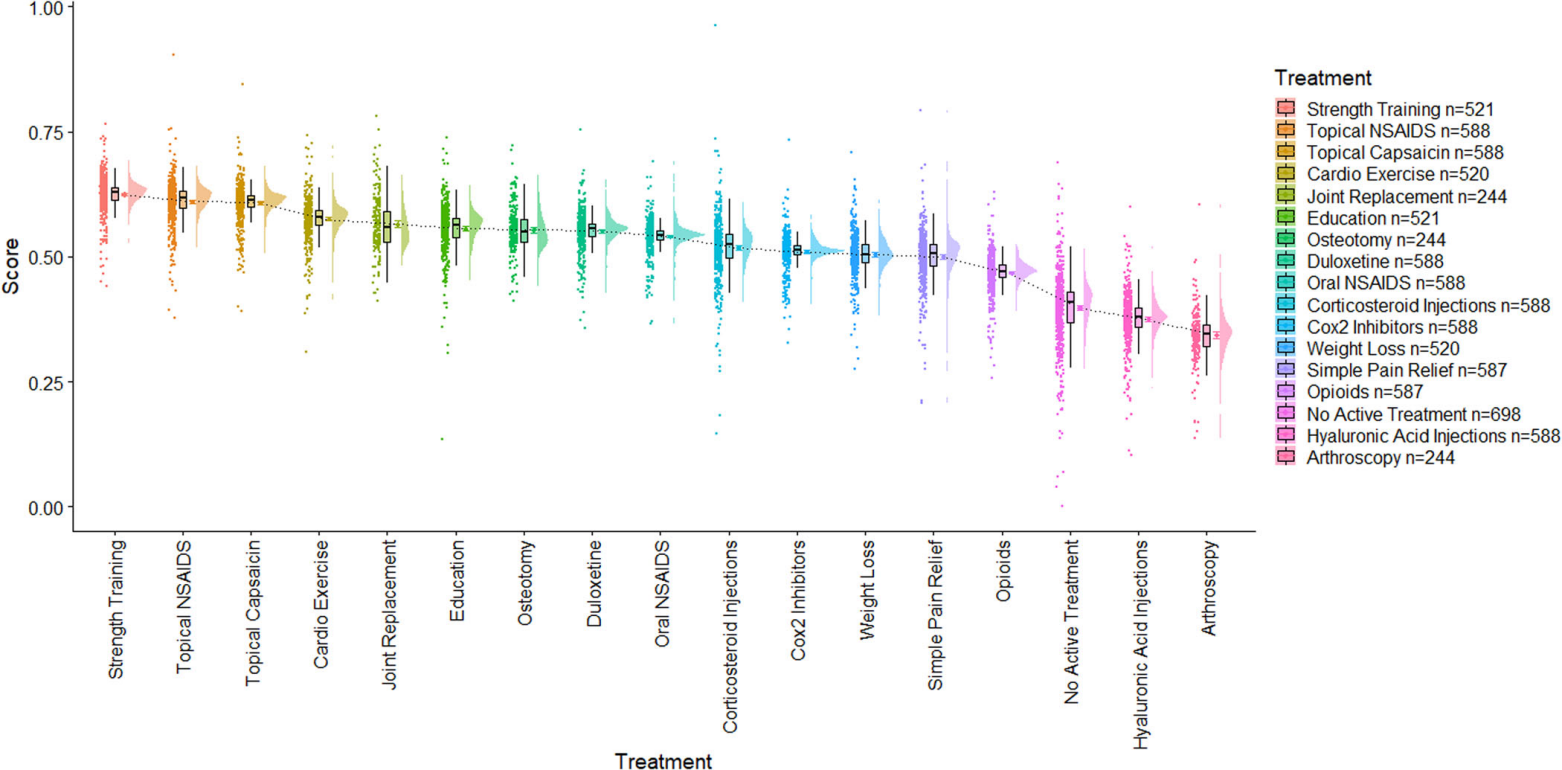
Raincloud plots of short‐form treatment scores from the least to the most preferred treatment

**Figure 4:**
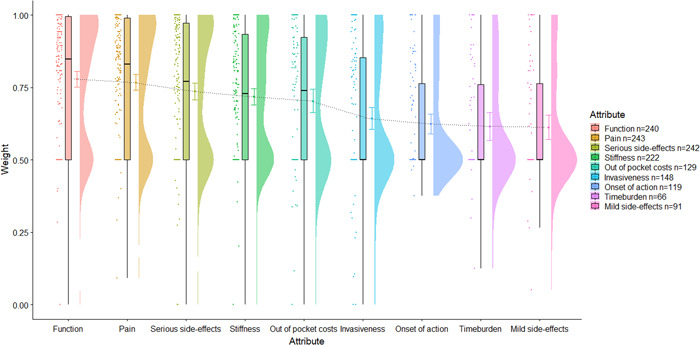
Raincloud plots of long‐form respondents determined importance weights for treatment attributes with number of respondents who chose to include each attribute

**Figure 5:**
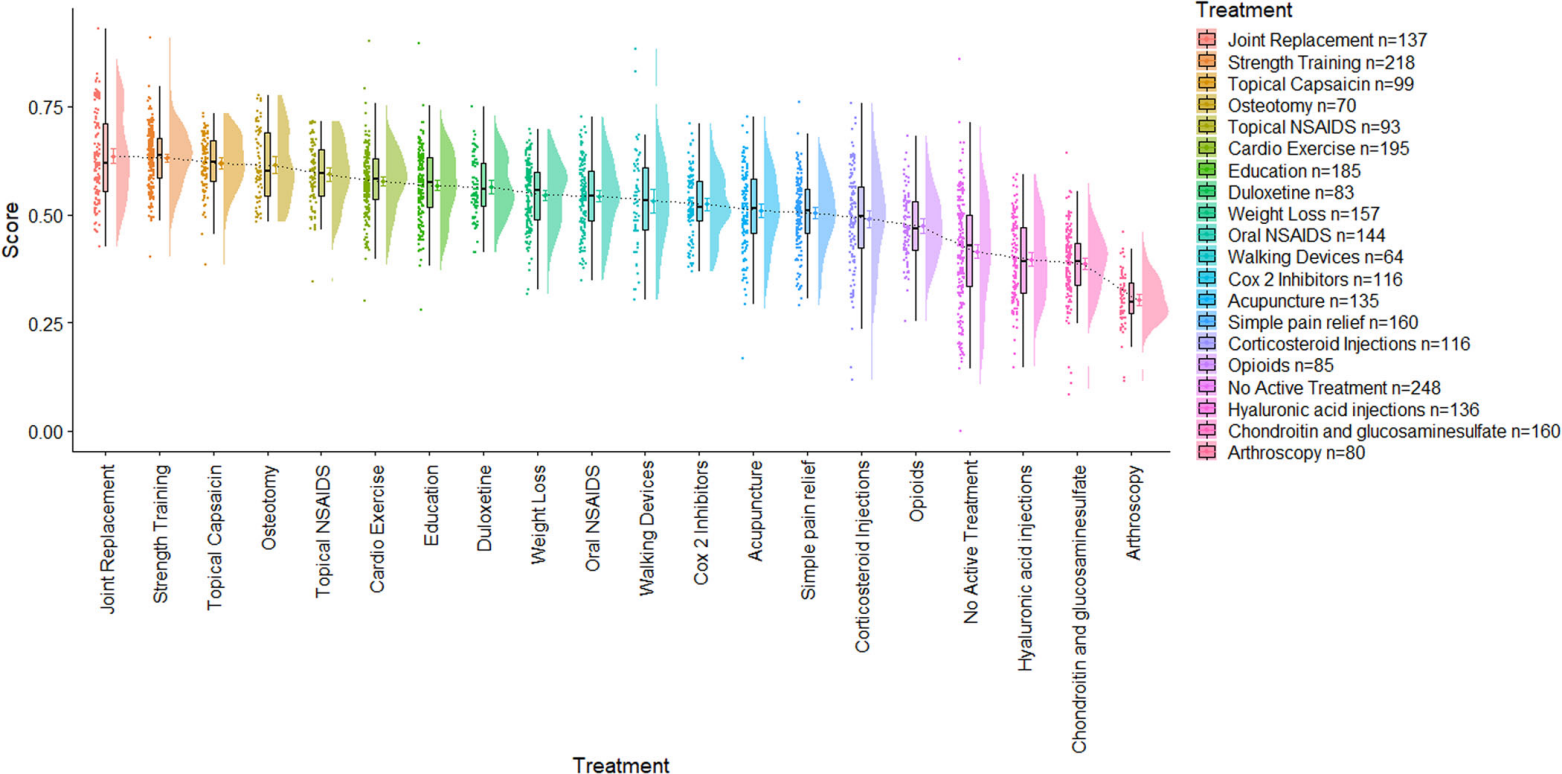
Raincloud plots of long‐form treatment scores from the least to the most preferred treatment

A first aim was to observe measures of central tendency and distribution of respondent importance weights and treatment scores. To this end, we utilized raincloud plots (Figures 2, 3, 4, 5) that visualize, in this case from left to right, (a) individual data points; (b) boxplots representing the median, interquartile range (IQR; the edges of the box) and total range to 1.5*IQR (the whiskers); (c) the mean and 95% confidence intervals; and (d) a split‐half violin plot representing the frequency distribution.^27^ These plots were chosen for presenting attribute weights and treatment scores as, in addition to measures of central tendency, they also provide a visual representation of the frequency distribution of the importance weights and, subsequently, the treatment scores. We also measured the frequency of selection of response options for the outcome questions. Pearson's correlation coefficients were used to ascertain whether difficulty of use was related to age. Analysis of variance was used to test whether there were significant differences in the length of time spent on the survey between people who did not change any of the importance weights versus those who did change the weights.

## 4 RESULTS

A total of 1158 self‐selected respondents consented to participate, with 625 continuing to the end of the short form. Out of these 625 respondents, 521 chose to begin the long form, with 180 completing the survey.

### 4.1 Short‐form Annalisa DA

A total of 561 (57.0%) respondents indicated that they would like to try ‘Lifestyle Change and Medicines’; 178 (18.1%) indicated that they would like to try ‘Medicines and Surgery’; 151 (15.3%) indicated that they would like to try ‘Lifestyle Changes and Surgery’; and 94 (9.6%) indicated that they would like to try ‘Medicines only’.

### 4.2 Attribute weights

Overall, serious side effects and function had the highest mean weights, and out‐of‐pocket costs and mild side effects had the lowest mean weights (see Figure 2). An unexpected finding was that 120 (13.5%) respondents weighed all treatment attributes as maximally important (10 out of 10).

### 4.3 Generated treatment scores

Overall, strength training had the highest mean score, and arthroscopy had the lowest mean score (see Figure 3).

### 4.4 Short‐form outcomes

Most respondents were not very surprised with their results: 197 (36.0%) reported being *not at all* surprised at the result, and similarly, 159 (29.1%) reported only feeling *slightly* surprised. Fewer people selected *fairly* (112, 20.5%), *very* (71, 13.0%) and *extremely* (8, 1.5%) surprised.

Most respondents (366, 67.5%) reported that the decision aid had not changed their view about treatment, although a substantial proportion reported that it had (176, 32.5%). Most respondents (350, 62.1%) stated that it was *very likely* that they would visit their GP or specialist in the next 6 months to talk about their treatment options, and only a minority reported *likely* (126, 23.1%%) or *not likely* (70, 12.8%).

### 4.5 Long‐form Annalisa DA

Respondent characteristics for the long‐form version are shown in Table 1. For treatment options, 262 (91.9%) respondents selected *lifestyle options and devices*; 205 (84.4%) selected *Medicines*; 183 (78.9%) selected *alternative medicines or complementary therapies*; and 162 (74.0%) selected *surgical interventions*.

**Table 1:** Long‐form respondent characteristics

	*n*	%
Age		
0–49	45	13.9
50–59	107	33.0
60–69	107	33.0
70+	65	20.1
Which joints are most affected		
Knee only	87	26.9
Primarily knee and other joints	114	35.2
Hip only	49	15.1
Hip and other joints	74	22.8
Diagnoses		
None	104	32.1
High blood pressure	81	25.0
Heart disease	15	4.6
Diabetes	19	5.9
Kidney disease	7	2.2
Liver disease	2	0.6
Peptic ulcer	4	1.2
Intestinal bleeding	3	0.9
Indigestion	27	8.3
Depression	30	9.4
Anaemia	6	1.9
Cancer	16	4.9
Back pain	108	33.3

Weights and, thus, scores did not change significantly across the three instances of the long‐form Annalisa DA (see Appendix SB for the exact scores for each instance). Overall, ‘function’ had the highest mean weight, and ‘mild side effects’ had the lowest mean weight. Importance weights and treatment scores from the first instance are shown in Figures 4 and 5.

### 4.6 Long‐form outcomes

Overall, most respondents (118, 63.8%) who completed the survey rated their experience using the tool as positive. Only 14 (7.6%) reported having a negative experience with the tool, and 62 (28.6%) reported having neither a positive nor a negative experience. Most respondents were not very surprised with their results: 62 (33.9%) reported being *not at all* surprised at the result, and similarly, 62 (33.9%) reported only feeling *slightly* surprised. As with the short form, fewer people selected *fairly* (38, 20.8%), *very* (17, 9.3%) and *extremely* (4, 2.2%). Most respondents (112, 61.5%) reported that the decision aid had not changed their view about treatment, although, as with the short form, a substantial proportion reported that it had (70, 38.5%). Most respondents (113, 62.1%) stated that it was *very likely* that they would visit their GP or specialist in the next 6 months to talk about their treatment options, and only a minority reported *likely* (49, 27.5%) or *not likely* (19, 10.4%). Importantly, a substantial number of respondents (89, 48.4%) reported that they thought the decision aid would help improve the quality of their future decisions, with a minority selecting that they thought it would not (33, 17.9%) or that they were unsure (62, 33.7%).

Overall, older people found the tool harder to use (see Table 2). When treated as a continuous variable, age correlated *r* = .32 with difficulty *rating the decision quality items*; *r* = .20 with *deciding on the length of the blue bars*; *r* = .28 with *understanding what was expected of me*; and *r* = .29 with *working out my result* (Pearson's bivariate correlations, all *p*'s < .01). However, as can be seen in Table 2, no age group had a mean difficulty score higher than the midpoint of the scale (i.e., 3; scale from 1 to 5).

**Table 2:** Mean respondent ratings of how difficult each aspect was to understand stratified by age group

	Age	*N*	Mean	SD
*Rating the decision quality items*	≤49	26	1.88	0.77
	50–59	49	2.18	0.76
	60–69	53	2.45	1.08
	≥70	29	2.86	1.03
*Deciding on the length of the blue bars*	≤49	29	2.10	0.82
	50–59	58	2.31	0.88
	60–69	61	2.54	1.07
	≥70	32	2.56	1.08
*Understanding what was expected of me*	≤49	29	2.00	0.89
	50–59	58	2.26	0.93
	60–69	62	2.65	1.11
	≥70	31	2.74	1.18
*Working out my result*	≤49	29	1.93	0.75
	50–59	58	2.43	1.05
	60–69	62	2.87	1.14
	≥70	31	2.77	1.28

*Note*: Scores range from 1 (very easy) to 5 (very hard).

An interesting finding was that 49 respondents (27.3%) did not change the weightings at all in any of the three instances of the long‐form DA. As such, these respondents were allocated the default 0.5 weighting for each attribute. Although it is possible that a proportion of people may actually weigh the attributes equally, we investigated the possibility that these people may not have been engaging with the tool. To this end, we analysed whether these people spent less time on the decision aids and appeared to skip through the video instructions. An initial analysis found that these respondents (mean = 3.04 min) were spending significantly less time on the ALs compared to respondents who did change weights (mean = 33.71 min). However, we noted the existence of two outliers in the latter group who had clearly left and returned later (13.95 and 69.45 h). After these two outliers had been eliminated, the mean time spent on the DAs for people who changed the weights was 5.88 min. This was still a significantly longer time than respondents who did not change the weights, *F*(1,224) = 9.292, *p* = .003, *η*² = 0.040. This difference implies that the people who did not change the weights may have been ‘rushing through’ the DAs. However, people who did not change the weights were just as likely to see the instructional videos through to completion, as indexed by whether they spent less time on those pages than the length of the video.

We were interested in investigating whether these respondents had simply not understood what they were meant to do. For the four questions measuring difficulty in using the tool, respondents who had not changed the weights tended to report that they found the tool *easier* to use. This finding was surprising as, by not changing the weights, these respondents appeared not to have a good understanding of the tool. We ran analyses to see if these people scored significantly differently on the questions about the usefulness of the tool, and for all of these outcomes, there were no significant differences between respondents who did or who did not change the weights. Again, this is surprising as the fact that they had not changed any weights should limit the usefulness of the tool.

## 5 DISCUSSION

The present study was conducted to develop and test the utility of a multicriteria PDA for OA in a self‐selected general population sample. In general, there was a positive response to the tool. Most respondents reported that using the tool was a positive experience, and a significant proportion reported that using the tool had changed their mind and that using the tool would improve the quality of their decision‐making. Overall, the findings suggest that this PDA may be of use to a substantial number of people with OA. These findings complement previously reported qualitative data from the use of this PDA,^26^ which found that many users reported that the tool enabled a sense of control over treatment decision‐making and provided them with a mechanism for more meaningful engagement with their clinician.

Unsurprisingly, serious side effects, pain and function were rated as the most important treatment outcomes. The relatively high importance placed on serious side effects is especially not surprising; previous research has also noted the high importance that people with OA place on avoiding serious side effects. In fact, Fraenkel et al.^28^ and Laba et al.^29^ found that patients with OA were often willing to accept lower treatment efficacy to lower the risk of serious side effects. Similarly, in a discrete‐choice experiment, Fraenkel and Fried ^30^ found that patients tended to prefer exercise as a treatment of choice because they were not willing to accept the risks of serious side effects. Importantly, treatments are often framed in terms of reducing pain and improving function, and the present findings reiterate that the values of the patients are not always aligned with those of the clinician.

Nevertheless, reducing pain and improving function were also weighed highly by respondents in the present study. The fact that these attributes were also the most included in the customizable long‐form AL also speaks to their importance for people with OA. This finding is in agreement with the findings of Ratcliffe et al.,^31^ who also found that pain, function and side effects played the biggest role in determining OA treatment preferences. In the present study, the importance of these factors likely contributed to the high treatment score for strength training: A treatment with reasonably high efficacy in reducing symptoms, but with a low risk of side effects.^32^, ^33^, ^34^ The fact that lifestyle changes scored relatively highly in the present study reiterates the importance of incorporating patient preferences into treatment decision‐making to avoid unnecessary invasive treatments. Nevertheless, more aggressive treatments such as joint replacement and osteotomy also scored relatively highly, corroborating previous research suggesting that these treatments may be appropriate for a substantial number of OA patients.^35^, ^36^ The key message is not that any specific treatment should be recommended across the board, but rather that individual differences in values should drive appropriate decision‐making.

OA is more prevalent in older people, and this demographic faces barriers to using online PDAs.^22^ These barriers are compounded by the fact that older people often report feeling comfortable with computers despite actually having a relatively low level of computer literacy.^22^ Older people are less likely to be involved in shared decision‐making, being more likely to accept without question the opinion of the treating physician.^37^, ^38^ Nonetheless, it is vital that age is not used as a barrier to eliciting patient preferences and that every effort is made to support informed decisions about appropriate treatment for their OA. Thus, it is imperative that barriers to the use of PDAs for older people are minimized. In the study reported here, we found that older age was related to increased difficulty in using the Annalisa decision tool. As previous research suggests that older people often prefer to make decisions between fewer options,^39^ future development of this PDA could involve further narrowing down the range of treatments that older people are interested in before they enter the Annalisa section where treatment options are presented. Similarly, as reduced decisional capacity in older people appears to be related to declines in working memory,^40^ further prompts reminding the user of how to use the PDA may also assist older people in using this PDA.

Nevertheless, there was no significant difference in age between people who did versus those who did not change any of the weights in the long‐form DA. Similarly, there were no differences in age between people who maximally weighted each treatment attribute. The lack of difference suggests that, although older people on average did report finding the tool harder to use, this did not necessarily lead to incomprehension of how to use the tool. Previous research suggests that older people can feel less confident about decisions that rely on cognitive ability^41^; however, the results of the present study did not suggest that older people were more likely to incorrectly engage with the tool. Furthermore, the fact that all age groups reported difficulty ratings below the midpoint of the scale suggests that the tool was reasonably easy to use for all ages.

### 5.1 Limitations

One limitation of the present study was the use of a self‐selected sample from an online website. As such, the weights and treatment scores obtained here do not establish any population norms around the relative weighting of treatment attributes. Furthermore, the online nature of the study meant that diagnoses of knee or hip OA were unable to be confirmed by X‐ray or clinician, which entails the possibility that some respondents may not actually have had OA. It should also be noted that the tool was not customized according to patient characteristics such as symptom burden and whether previous treatments had been tried.

Furthermore, the significant dropout rate within each version of the study (short and long forms) also presents an issue for the acceptability of the tool used in this context. Participants who dropped out of the study may be less likely to have a positive experience, and this might have biased the outcomes pertaining to the usefulness of the tool. Although a substantial proportion of respondents reported that using the PDA had changed their views on treatment and that they thought using the PDA would increase the quality of the treatment decisions, another limitation of the present study was that there is no way of knowing if the use of the PDA had any effect on actual treatment decisions or whether respondents would follow up to discuss their treatment options with their GP or specialist.

A significant proportion of respondents either weighed all attributes as maximally important or did not change the weights from the default of 0.5 in the long form (this would not apply for the short‐form as the initial weights in the short form were automatically inputted from earlier multiple‐choice questions; thus, the weights upon arriving in the short‐form AL had already been inputted). Although it is possible that such responding reflects the true preferences of these people, it seems, *prima facie*, unlikely that all of these respondents truly value each treatment attribute as equally important. Two more plausible explanations are that (a) many of these respondents did not know what they were expected to do, or (b) understood but simply did not care to engage with the tool properly. Both of these possibilities would be mitigated in an in‐person clinical setting, but the former is a concern for the applicability of the tool as a stand‐alone online resource. However, it is surprising that these respondents did not report finding the tool more difficult to use or less useful. Nevertheless, caution should be exercised when interpreting the rankings of treatments presented here as these scores may have been biased by the inclusion of respondents who did not change the importance weights from the default position of 0.5 in the long form. The significant number of respondents who did not change the weightings in the long form potentially represents a challenge for the acceptability of the tool in its current form. Although it is possible that the default positions represented the true preferences of some proportion of these respondents, it is also possible that these respondents were not engaging with the tool correctly.

## 6 CONCLUSION

The present study introduces a multicriteria PDA for OA and demonstrates its usefulness in a self‐selected sample of people with OA. In both the short and long forms of the survey, *function*, *serious side effects* and *pain* were the three most important treatment attributes. The tool appeared to be useful to many participants, with most respondents reporting a positive experience using the tool, and over a third reporting that the tool had changed their view on treatment. Nevertheless, a high dropout rate suggests that many users of the tool disengaged from it. Older respondents reported greater difficulty using the tools, suggesting that further work is needed to optimize the tool for older people. Further iterations of the PDA should be simplified with enhanced personalization to the individual to get the most out of an evidence‐ and preference‐based approach to informed medical decision‐making. Future research with this tool could investigate the relative importance of customization versus length in determining the usefulness of the tool. Further testing is also needed into the potential effects of the tool on quality of life and functioning, as well as evaluation from the healthcare professionals working with people using this PDA.

## AUTHOR CONTRIBUTIONS

All authors except Sam G. Moreton were involved in the background and design of the study. The construction of the tool was led by Sally Wortley. Sam G. Moreton and Glenn Salkeld wrote the first draft of the manuscript. Data analysis was conducted by Sam G. Moreton and Glenn Salkeld. All authors provided critical feedback and helped shape the analysis and manuscript.

## CONFLICTS OF INTEREST

David J. Hunter provides consulting advice to Pfzier, Lilly, Merck Serono and TLCBio outside the submitted work.

## Supporting information

Supporting information.

Supporting information.

## Data Availability

Data are available upon request.
