# Supplementary material for: The development and utility of a multicriteria patient decision aid for people contemplating treatment for osteoarthritis
Source: Health Expect. 2022 Aug 30;25(6):2775–85. doi: 10.1111/hex.13505 (PMC9700162; doi:10.1111/hex.13505)
Supplement: Supplementary file 1 — Supporting information. [file HEX-25--s002.docx]

**SUPPLEMENTARY MATERIAL FOR AL MCDA: person-important outcomes and effectiveness of treatment options**

1. **BACKGROUND**

Decision aids seek to assist individuals make informed value based decision about choices. This is achieved by helping each person determine what is important to them in the decision as well as displaying the relevant options available. The evidence presented on these options should reflect the best available and contain information on both the harms and benefits of each option.

Annalisa© (AL) is a web-based decision-aid tool grounded in multi-criteria decision analysis (MCDA). Three sections are included in this tool which cover; the individual’s values and preferences, available treatment options and the evidence base on the performance of each option. AL uses a simple expected value algorithm to calculate a score for each option by taking into account the individual's preferences and the evidence of the performance of each option on each criterion. By combining this evidence the best course of action (elicited in a graphical way at the point of decision), for each patient will be identified on the basis of quantified scores for each option.

- 1. **Topical Development Sequence**


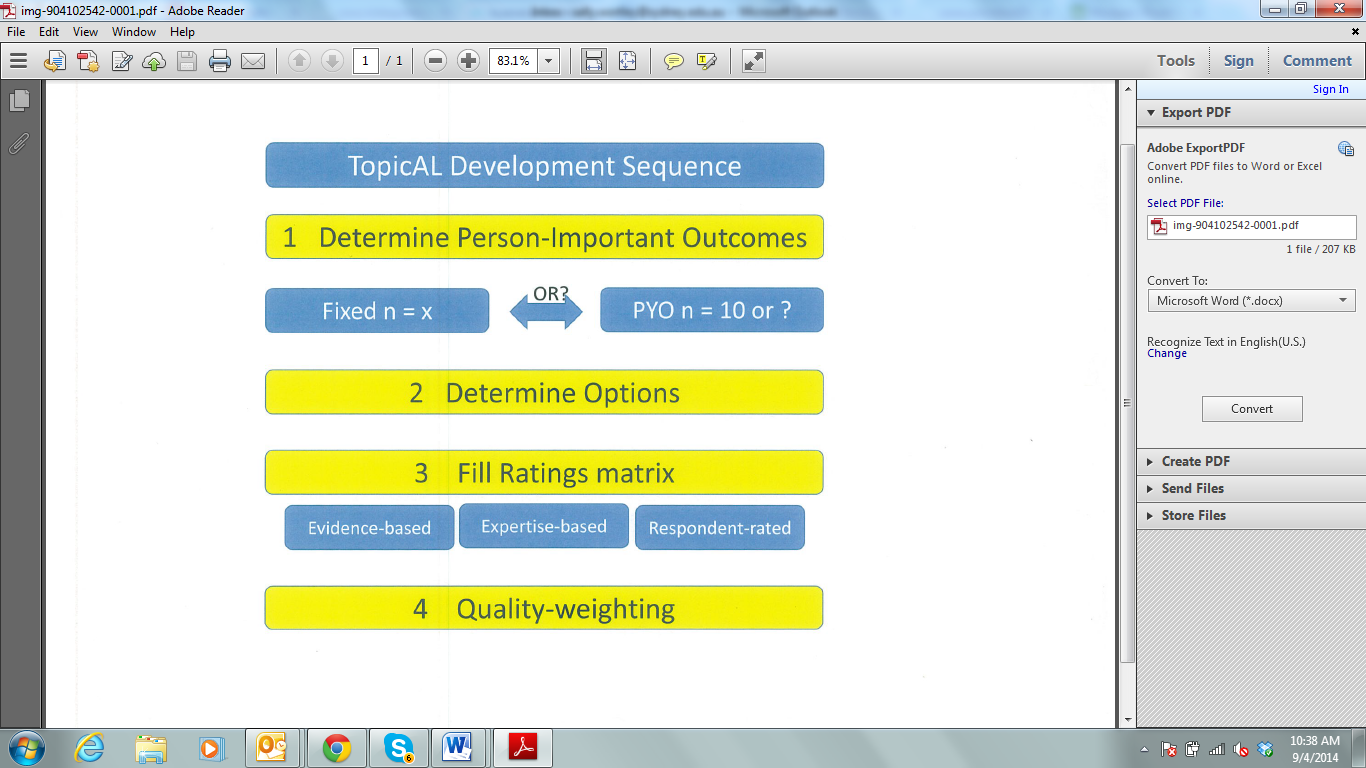


This document is concerned with identifying literature to inform:

- a list of person-important outcomes and
- performance of each listed treatment options (rating matrix)

1. **DeFINING THE SCOPE**

This will be topic specific and will determine the treatment options and person important outcomes for the decision aid. Preliminary information about treatment options and person important outcomes may be identified at this stage. For the Osteoarthritis AL, the scope will be determined by the research team.

1. **PERSON-IMPORTANT OUTCOMES**
   1. **Types of studies**

We will include studies which report on the population of interest and include information on values, preferences or factors that are important to individuals when making treatment decisions. As such studies will need to address the relevant treatment options. Included studies may be those that provide revealed or stated preference data.

Our focus will be on study designs such as:

- qualitative reports based on focus groups, in-depth interviews
- Qualitative syntheses
- Literature reviews
- Decision aid evaluations
- Discrete choice experiments (DCE)

- 1. **Types of outcome measures**

We will include studies which report on the following outcomes:

- Factors that are important to individuals in making decision about treatment
- Attributes that have been included in DCEs or decision aids looking at treatment
- Values and/or preferences that have been identified in the literature
  1. **Search methods for identification of studies**

We will search the following electronic databases:

- Embase.com (Medline, Embase),
- PsycINFO,
- Econlit, SocoFile
- Google Scholar

Should a recent systematic review be identified at the start of the search, the remaining search will focus on identifying any additional evidence reported beyond the publication date of the identified report.

For the Osteoarthritis AL – studied need to report on individuals with osteoarthritis (or suspected osteoarthritis) of the knee and hip, include treatment options covered by myjointpain website, report on patients reported outcome measures in respect to treatment decision-making. Only full text studies and English language studies will be included.

Literature searching will be based on a combination of text words and MESH words that includes the following: patient reported outcome measures, choice behavior, decision making, patient satisfaction, patient participation, consumer satisfaction, patient compliance and attitude to health and osteoarthritis.

Studies will be excluded if they do not report patient populations and interventions and outcomes as described above.

- 1. **Data collection and analysis**

Once a literature search has been undertaken the references will be downloaded into reference management software. Inclusion and exclusion criteria will then be applied to the abstracts based on population, study design and outcomes.

Articles that meet the criteria were then retrieved and data extracted using either context analysis or thematic analysis to construct a list of person important outcomes. This will then be presented to the research team for discussion.

- 1. **RESULTS**

The literature search was conducted in December 2013 and identified 386 non-duplicate studies. Twenty-two studies meet the inclusions criteria. The majority of studies focused on preferences of individuals undergoing or contemplating joint replacement surgery. Almost all of the 22 studies noted the importance of improvement in clinical outcomes such as pain and function.

A set of twelve patient important outcomes were identified following the literature review, these included reduction in pain, improvement in function and stiffness, avoidance of side effects (mild and serious), invasiveness, time burden, duration of action, out of pocket expenses, recovery time, emotional well-being, duration of pain relief. Following discussions with the research team, this was reduced to nine outcomes. This was based on the opinion that the estimates underpinning the three outcomes (recovery time, emotional well-being, duration of pain relief) were not as relevant to all OA patients and/or estimates would be difficult to identify.

1. **EVIDENCE FOR RATINGS MATRIX**

To identify the best scientific evidence, the literature must be recent and relate to the available treatment options and their consequences.

- 1. **Types of studies**

Studies will need to be comparative given the tool compares one treatment to another or to placebo (in the case of multiple options). In order to reduce systematic and random error, emphasis will be placed on identifying evidence from the following sources:

- clinical guidelines,
- systematic reviews,
- meta-analyses or
- health technology assessment reports on the topic of interest.
- Greater priority will be given to guidelines, systematic reviews and HTA reports based on evidence standards equivalent to NHMRC Grades I and II and where sufficient methodology has been reported to identify the process and analysis of the literature.

Cost effectiveness models may also be considered on a topic if they have been informed by recent literature review.

- 1. **Types of outcome measures**

Studies need to report on relevant person important outcomes relevant to treatment options

Ideally probabilities should be reported or be able to be calculated.

- 1. **Search methods for identification of studies**

For the Osteoarthritis AL the following sources were identified as sources of evidence of effectiveness:

Initial scoping search:

- Clinical practice guidelines database (<http://www.guideline.gov/>),
- A focused search of websites of key health care agencies involved in clinical guidelines production: NHMRC (Australia), UK (NICE, SIGN), US (AHRQ) and Canada (CADTH)
- Specialist societies relevant to the topic.
- Cochrane library, including the Cochrane database of systematic reviews, Database of abstracts of reviews of effects (DARE) and the HTA database/CRD database, NHS EED and HEED databases. This search will focus on high level literature i.e. systematic reviews, meta-analysis and trials

Additional search sources if required:

- Embase.com (Medline, Embase) will also be searched using similar terms to that for the Cochrane library
- Google Scholar

Literature searches will be restricted to English language only. Preference will also be given to studies in location in a region with a comparative health system to Australia.

Should a recent high-quality guideline/systematic review or HTA report be identified at the start of the search, the remaining search will focus on identifying any additional high level evidence reported beyond the publication date of the identified report.

Literature searching will be based on a combination of text words and MESH words around osteoarthritis for initial scoping search. This will then be widened depending on any gaps in the evidence base for the treatment options. References lists of any guidelines will also be used.

- 1. **Data collection and analysis**

Once a literature search has been undertaken the references will be downloaded into references management software. Inclusion and exclusion criteria will then be applied to the abstracts based on population, study design and outcomes.

Articles that meet the criteria were then retrieved and data extracted using either context analysis or thematic analysis to construct a list of person important outcomes. This will then be presented for comment.

- 1. **RESULTS**

The literature search was conducted in April 2014. From the initial search, three guidelines were identified:

- Osteoarthritis Research Society International (OARSI) Guidelines for non-surgical management of osteoarthritis.
- NICE Guidelines on Osteoarthritis <https://www.nice.org.uk/guidance/CG177>
- American Academy of Surgeons (AAOS) Clinical Practice Guideline on Treatment of Osteoarthritis of the Knee <https://aaos.org/quality/quality-programs/lower-extremity-programs/osteoarthritis-of-the-knee/>

These guidelines were used as the major source of evidence for the Osteoarthritis AL. Hand searching, supplementary searching identified a further 51 studies relevant to the Osteoarthritis AL (see Appendix A). These additional studies covered areas not reported on in the above guidelines.

Data extraction of relevant points were extracted and presented to the research team for discussion. A final spreadsheet was created with all the evidence points.

**Appendix A: Additional references for the evidence matrix**

Bannuru RR, Dasi UR, McAlindon TE: **558 Reassessing the role of acetaminophen in osteoarthritis: systematic review and meta-analysis.** *Osteoarthritis and Cartilage* 2010, **18:**S250.

Bannuru RR, Schmid CH, Kent DM, Vaysbrot EE, Wong JB, McAlindon TE: **Comparative Effectiveness of Pharmacologic Interventions for Knee Osteoarthritis: A Systematic Review and Network Meta-analysis.** *Annals of internal medicine* 2015, **162:**46-54.

Bellamy N, Campbell J, Welch V, Gee TL, Bourne R, Wells GA: **Intraarticular corticosteroid for treatment of osteoarthritis of the knee (Review).** 2009.

Brand E, Nyland J, Henzman C, Mcginnis M: **Arthritis self-efficacy scale scores in knee osteoarthritis: a systematic review and meta-analysis comparing arthritis self-management education with or without exercise.** *journal of orthopaedic & sports physical therapy* 2013, **43:**895-910.

Brouwer RW, Huizinga MR, Duivenvoorden T, van Raaij TM, Verhagen AP, Biermaeinstra S et al.: Osteotomy for treating knee osteoarthritis. The Cochrane Library 2014.

Chappell AS, Ossanna MJ, Liu-Seifert H, Iyengar S, Skljarevski V, Li LC, Bennett RM, Collins H: **Duloxetine, a centrally acting analgesic, in the treatment of patients with osteoarthritis knee pain: a 13-week, randomized, placebo-controlled trial.** *Pain* 2009, **146:**253-260.

Chodosh J, Morton SC, Mojica W, Maglione M, Suttorp MJ, Hilton L, Rhodes S, Shekelle P: **Meta-analysis: chronic disease self-management programs for older adults.** *Annals of internal medicine* 2005, **143:**427-438.

Christensen R, Bartels EM, Astrup A, Bliddal H: **Effect of weight reduction in obese patients diagnosed with knee osteoarthritis: a systematic review and meta-analysis.** *Annals of the rheumatic diseases* 2007, **66:**433-439.

Christensen R, Henriksen M, Leeds AR, Gudbergsen H, Christensen P, S++rensen TJ, Bartels EM, Riecke BF, Aaboe J, Frederiksen R: **The effect of weight maintenance on symptoms of knee osteoarthritis in obese patients: 12 month randomized controlled trial.** *Arthritis Care & Research* 2014.

Coleman S, Briffa NK, Carroll G, Inderjeeth C, Cook N, McQuade J: **A randomised controlled trial of a self-management education program for osteoarthritis of the knee delivered by health care professionals.** *Arthritis Res Ther* 2012, **14:**R21.

Derry S, Moore RA, Rabbie R: **Topical NSAIDs for chronic musculoskeletal pain in adults.** *The Cochrane Library* 2012.

Du S, Yuan C, Xiao X, Chu J, Qiu Y, Qian H: **Self-management programs for chronic musculoskeletal pain conditions: a systematic review and meta-analysis.** *Patient education and counseling* 2011, **85:**e299-e310.

Fary RE, Carroll GJ, Briffa TG, Briffa NK: **The effectiveness of pulsed electrical stimulation in the management of osteoarthritis of the knee: Results of a double−blind, randomized, placebo−controlled, repeated−measures trial.** *Arthritis & Rheumatism* 2011, **63:**1333-1342.

Frakes EP, Risser RC, Ball TD, Hochberg MC, Wohlreich MM: **Duloxetine added to oral nonsteroidal anti-inflammatory drugs for treatment of knee pain due to osteoarthritis: results of a randomized, double-blind, placebo-controlled trial.** *Current Medical Research & Opinion* 2011, **27:**2361-2372.

Fransen M, McConnell S, Hernandez-Molina G, Reichenbach S: **Land-based exercise for osteoarthritis of the hip: updated systematic review and meta-analysis.** *Osteoarthritis and Cartilage* 2014, **22:**S51.

Hagino T, Ochiai S, Watanabe Y, Senga S, Wako M, Ando T, Sato E, Haro H: **Complications after arthroscopic knee surgery.** *Archives of orthopaedic and trauma surgery* 2014, **134:**1561-1564.

Hochberg MC, Wohlreich M, Gaynor P, Hanna S, Risser R: **Clinically relevant outcomes based on analysis of pooled data from 2 trials of duloxetine in patients with knee osteoarthritis.** *The Journal of rheumatology* 2012, **39:**352-358.

Jansen MJ, Viechtbauer W, Lenssen AF, Hendriks EJ, de Bie RA: **Strength training alone, exercise therapy alone, and exercise therapy with passive manual mobilisation each reduce pain and disability in people with knee osteoarthritis: a systematic review.** *Journal of physiotherapy* 2011, **57:**11-20.

Jones A, Silva PG, Silva AC, Colucci M, Tuffanin A, Jardim JR *et al*.: **Impact of cane use on pain, function, general health and energy expenditure during gait in patients with knee osteoarthritis: a randomised controlled trial.** *Annals of the rheumatic diseases* 2011,

Juhl C, Christensen R, Roos EM, Zhang W, Lund H: **Impact of Exercise Type and Dose on Pain and Disability in Knee Osteoarthritis: A Systematic Review and Meta-Regression Analysis of Randomized Controlled Trials.** *Arthritis & rheumatology* 2014, **66:**622-636.

Kemp JL, MacDonald D, Collins NJ, Hatton AL, Crossley KM: **Hip Arthroscopy in the Setting of Hip Osteoarthritis: Systematic Review of Outcomes and Progression to Hip Arthroplasty.** *Clinical Orthopaedics and Related Research-«* 2014,1-19.

Kirkley A, Birmingham TB, Litchfield RB, Giffin JR, Willits KR, Wong CJ, Feagan BG, Donner A, Griffin SH, D'Ascanio LM: **A randomized trial of arthroscopic surgery for osteoarthritis of the knee.** *New England Journal of Medicine* 2008, **359:**1097-1107.

Kivitz A, Eisen G, Zhao WW, Bevirt T, Recker DP: **Randomized placebo-controlled trial comparing efficacy and safety of valdecoxib with naproxen in patients with osteoarthritis.** *Journal of family practice* 2002, **51:**530-537.

Kosuwon W, Sirichatiwapee W, Wisanuyotin T, Jeeravipoolvarn P, Laupattarakasem W: **Efficacy of symptomatic control of knee osteoarthritis with 0.0125% of capsaicin versus placebo.** *Medical journal of the Medical Association of Thailand* 2010, **93:**1188.

Kowalczuk M, Bhandari M, Farrokhyar F, Wong I, Chahal M, Neely S, Gandhi R, Ayeni OR: **Complications following hip arthroscopy: a systematic review and meta-analysis.** *Knee Surgery, Sports Traumatology, Arthroscopy* 2013, **21:**1669-1675.

Laba TL, Brien Ja, Fransen M, Jan S: **Patient preferences for adherence to treatment for osteoarthritis: the MEdication Decisions in Osteoarthritis Study (MEDOS).** *BMC musculoskeletal disorders* 2013, **14:**160

Lambert RG, Hutchings EJ, Grace MG, Jhangri GS, ConnerΓÇÉSpady B, Maksymowych WP: **Steroid injection for osteoarthritis of the hip: A randomized, double−blind, placebo controlled trial.** *Arthritis & Rheumatism* 2007, **56:**2278-2287.

Laslett L, Jones G: **Capsaicin treatment for osteoarthritis pain: a meta-analysis.** *Osteoarthritis and Cartilage* 2014, **22:**S422.

Lee C, Hunsche E, Balshaw R, Kong SX, Schnitzer TJ: **Need for common internal controls when assessing the relative efficacy of pharmacologic agents using a metaΓÇÉanalytic approach: Case study of cyclooxygenase selective inhibitors for the treatment of osteoarthritis.** *Arthritis Care & Research* 2005, **53:**510-518.

Lun V, Marsh A, Bray R, Lindsay D, Wiley P: **Efficacy of Hip Strengthening Exercises Compared With Leg Strengthening Exercises on Knee Pain, Function, and Quality of Life in Patients With Knee Osteoarthritis.** *Clinical journal of sport medicine: official journal of the Canadian Academy of Sport Medicine* 2015.

Manyanga T, Froese M, Zarychanski R, Abou-Setta A, Friesen C, Tennenhouse M, Shay BL: **Pain management with acupuncture in osteoarthritis: a systematic review and meta-analysis.** *BMC complementary and alternative medicine* 2014, **14:**312.

March L, Cross M, Tribe K, Lapsley H, Courtenay B, Brooks P: **Cost of joint replacement surgery for osteoarthritis: the patients' perspective**. The Journal of rheumatology 2002, 29: 1006-1014.

Mason L, Moore RA, Derry S, Edwards JE, McQuay HJ: **Systematic review of topical capsaicin for the treatment of chronic pain.** *Bmj* 2004, **328:**991.

McKnight PE, Kasle S, Going S, Villanueva I, Cornett M, Farr J, Wright J, Streeter C, Zautra A: **A comparison of strength training, self−management, and the combination for early osteoarthritis of the knee.** *Arthritis Care & Research* 2010, **62:**45-53.

Medical Advisory Secretariat (MAS). **Total knee replacement: an evidence-based analysis**. 5(9). 2005. Ontario Health Technology Assessment Series.

Negm A, Lorbergs A, MacIntyre NJ: **Efficacy of low frequency pulsed subsensory threshold electrical stimulation vs placebo on pain and physical function in people with knee osteoarthritis: systematic review with meta-analysis.** *Osteoarthritis and Cartilage* 2013, **21:**1281-1289.

Quicke JG, Foster NE, Thomas MJ, Holden MA: **Is long-term physical activity safe for older adults with knee pain?: a systematic review.** *Osteoarthritis and Cartilage* 2014, **22:**S11-S12

Reichenbach S, Sterchi R, Scherer M, Trelle S, Buergi E, Buergi U, Dieppe PA, Juêni P: **Meta-analysis: chondroitin for osteoarthritis of the knee or hip.** *Annals of internal medicine* 2007, **146:**580-590.

Rejeski WJ, Focht BC, Messier SP, Morgan T, Pahor M, Penninx B: **Obese, older adults with knee osteoarthritis: weight loss, exercise, and quality of life.** *Health Psychology* 2002, **21:**419.

Risser RC, Hochberg MC, Gaynor PJ, DΓÇÖSouza DN, Frakes EP: **Responsiveness of the Intermittent and Constant Osteoarthritis Pain (ICOAP) scale in a trial of duloxetine for treatment of osteoarthritis knee pain.** *Osteoarthritis and Cartilage* 2013, **21:**691-694.

Rutjes AW, Nesch E, Sterchi R, Kalichman L, Hendriks E, Osiri M, Brosseau L, Reichenbach S, J++ni P: **Transcutaneous electrostimulation for osteoarthritis of the knee.** *The Cochrane Library* 2009.

Salzler MJ, Lin A, Miller CD, Herold S, Irrgang JJ, Harner CD: **Complications after arthroscopic knee surgery.** *The American journal of sports medicine* 2014, **42:**292-296.

Segal L, Day SE, Chapman AB, Osborne RH: **Can we reduce disease burden from osteoarthritis?** *Medical Journal of Australia (MJA)* 2004, **180:**S11-S17.

Shan L, Shan B, Suzuki A, Nouh F, Saxena A: Intermediate and Long-Term Quality of Life After Total Knee Replacement. The Journal of Bone & Joint Surgery 2015, 97:156-168.

Singh JA, Noorbaloochi S, MacDonald R, Maxwell LJ: Chondroitin for osteoarthritis. The Cochrane Library 2015.

Stam WB, Jansen JP, Taylor SD: **Efficacy of etoricoxib, celecoxib, lumiracoxib, non-selective NSAIDs, and acetaminophen in osteoarthritis: a mixed treatment comparison.** *The open rheumatology journal* 2012, **6:**6.

Towheed T, Maxwell L, Judd M, Catton M, Hochberg MC, Wells GA: **Acetaminophen for osteoarthritis.** *The Cochrane Library* 2006.

Wandel S, Juni P, Tendal B, Nesch E, Villiger PM, Welton NJ, Reichenbach S, Trelle S: **Effects of glucosamine, chondroitin, or placebo in patients with osteoarthritis of hip or knee: network meta-analysis.** *Bmj* 2010, **341**.

Yan JH, Gu WJ, Sun J, Zhang WX, Li BW, Pan L: **Efficacy of Tai Chi on pain, stiffness and function in patients with osteoarthritis: a meta-analysis.** *PLoS One* 2013, **8:**e61672.

Zeng C, Yang T, Deng Z, Yang Y, Zhang Y, Lei G: **Electrical stimulation for pain relief in knee osteoarthritis: systematic review and network meta-analysis.** *Osteoarthritis and Cartilage* 2014.
