# Supplementary material for: The development and utility of a multicriteria patient decision aid for people contemplating treatment for osteoarthritis
Source: Health Expect. 2022 Aug 30;25(6):2775–85. doi: 10.1111/hex.13505 (PMC9700162; doi:10.1111/hex.13505)
Supplement: Supplementary file 2 — Supporting information. [file HEX-25--s001.docx]

# Appendix B

Appendix B Table 1. Mean long-form respondents determined importance weights for treatment attributes.

|  | N | | |  | Mean | | |
| --- | --- | --- | --- | --- | --- | --- | --- |
|  | 1^st^ | 2^nd^ | 3^rd^ |  | 1^st^ | 2^nd^ | 3^rd^ |
| Function | 240 | 227 | 166 |  | .78 | .78 | .79 |
| Pain | 243 | 230 | 167 |  | .77 | .78 | .79 |
| Serious Side Effects | 242 | 230 | 167 |  | .74 | .74 | .75 |
| Stiffness | 222 | 210 | 153 |  | .72 | .73 | .72 |
| Out of pocket costs | 129 | 123 | 97 |  | .70 | .71 | .73 |
| Invasiveness | 148 | 140 | 103 |  | .64 | .65 | .63 |
| Onset of action | 119 | 115 | 79 |  | .62 | .62 | .64 |
| Time Burden | 66 | 64 | 46 |  | .62 | .61 | .61 |
| Mild Side Effects | 91 | 85 | 67 |  | .61 | .62 | .59 |

Appendix B Table 2. Mean long-form scores that were obtained for each treatment.

|  | N | | |  | Mean | | |
| --- | --- | --- | --- | --- | --- | --- | --- |
|  | 1^st^ | 2^nd^ | 3^rd^ |  | 1^st^ | 2^nd^ | 3^rd^ |
| Joint replacement | 137 | 134 | 94 |  | .63 | .63 | .62 |
| Strength training | 218 | 205 | 153 |  | .63 | .63 | .63 |
| Topical Capsaicin | 99 | 96 | 70 |  | .62 | .62 | .62 |
| Osteotomy | 70 | 69 | 47 |  | .61 | .61 | .60 |
| Topical NSAIDS | 93 | 91 | 64 |  | .59 | .59 | .59 |
| Cardio Exercise | 195 | 184 | 135 |  | .58 | .58 | .58 |
| Education | 185 | 173 | 134 |  | .57 | .57 | .57 |
| Duloxetine | 83 | 80 | 63 |  | .56 | .56 | .56 |
| Weight loss | 157 | 148 | 108 |  | .54 | .54 | .54 |
| Oral NSAIDs | 144 | 139 | 100 |  | .54 | .54 | .55 |
| Walking devices | 64 | 62 | 44 |  | .53 | .53 | .53 |
| Cox2 Inhibitors | 116 | 111 | 82 |  | .52 | .52 | .52 |
| Acupuncture | 135 | 125 | 97 |  | .51 | .52 | .51 |
| Simple pain relief | 160 | 155 | 110 |  | .50 | .50 | .50 |
| Corticosteroid Injections | 119 | 115 | 78 |  | .49 | .49 | .48 |
| Opioids | 85 | 81 | 60 |  | .47 | .48 | .48 |
| No active treatment | 248 | 235 | 171 |  | .41 | .42 | .42 |
| Hyaluronic acid injections | 136 | 131 | 92 |  | .40 | .39 | .39 |
| Chondroitin and glucosaminesulfate | 160 | 150 | 114 |  | .39 | .39 | .39 |
| Arthroscopy | 80 | 78 | 56 |  | .30 | .30 | .30 |

Appendix B Figure 1. Mean respondent scoring of time-burden of different treatment requirements


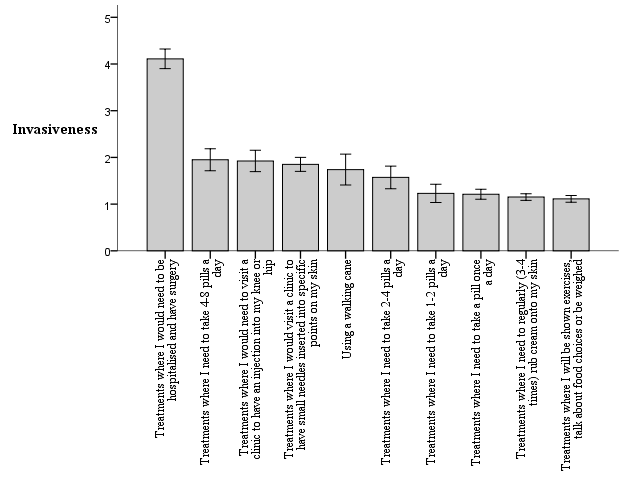


Appendix B Figure 2. Mean respondent scoring of time-burden of different treatment requirements


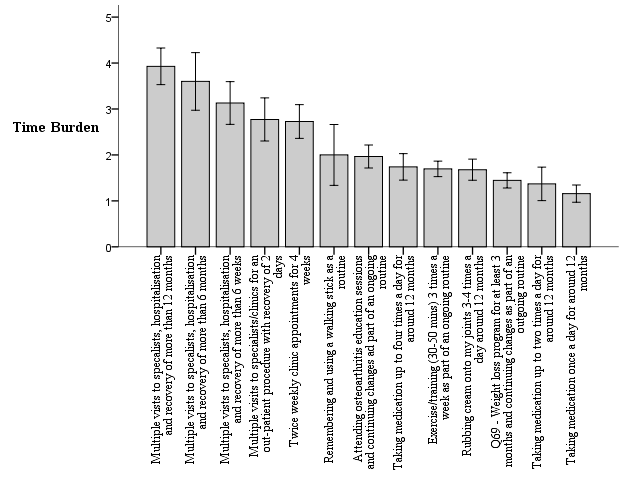


Appendix B Table 3. Respondent mean ratings of how difficult each aspect was to understand stratified by whether they changed the weights. Higher scores indicate more difficulty.

|  |  | N | Mean | SD |
| --- | --- | --- | --- | --- |
| *Rating the decision quality items* | Didn’t Change | 32 | 2.06 | 1.014 |
|  | Changed | 125 | 2.43 | .953 |
| *Deciding on the lengths of the blue bars* | Didn’t Change | 39 | 2.10 | .995 |
|  | Changed | 140 | 2.48 | .971 |
| *Understanding what was expected* | Didn’t Change | 39 | 2.08 | .957 |
|  | Changed | 140 | 2.54 | 1.075 |
| *Working out my result* | Didn’t Change | 39 | 2.38 | 1.161 |
|  | Changed | 140 | 2.62 | 1.122 |
